# Detection of autoantibodies to citrullinated BiP in rheumatoid arthritis patients and pro-inflammatory role of citrullinated BiP in collagen-induced arthritis

**DOI:** 10.1186/ar3520

**Published:** 2011-11-22

**Authors:** Hirofumi Shoda, Keishi Fujio, Mihoko Shibuya, Tomohisa Okamura, Shuji Sumitomo, Akiko Okamoto, Tetsuji Sawada, Kazuhiko Yamamoto

**Affiliations:** 1 Department of Allergy and Rheumatology, Graduate School of Medicine, the University of Tokyo, 7-3-1 Hongo, Bunkyo-ku, Tokyo, Japan; 2 Department of Rheumatology, Tokyo Medical University, 6-7-1, Nishisinjyuku, Sinjuku-ku, Tokyo, Japan

## Abstract

**Introduction:**

Anti-citrullinated protein/peptide antibodies (ACPAs) are highly specific to rheumatoid arthritis (RA) patients and are thought to have a close relationship with the pathogenesis of arthritis. Several proteins, including fibrinogen, vimentin, and alpha-enolase, were reported as ACPA-target antigens, and their importance in RA pathogenesis was widely proposed. We identified citrullinated immunoglobulin binding protein (citBiP) as another ACPA target in RA patients and examined its pro-inflammatory role in arthritis.

**Methods:**

We measured the levels of anti-citBiP, anti-BiP, and anti-cyclic citrullinated peptide (CCP) antibodies in the serum of RA patients (n = 100), systemic lupus erythematosus (SLE) patients (n = 60), and healthy controls (n = 30) using ELISA and immunoblotting. Epitope mapping was performed using 27 citBiP-derived peptides. In the mouse study, after DBA/1J mice were immunized with BiP or citBiP, serum titers of ACPAs were measured by ELISA and immunohistochemistry. The development of collagen-induced arthritis (CIA) was observed in BiP- or citBiP-pre-immunized mice.

**Results:**

The serum levels of anti-BiP and anti-citBiP antibodies were significantly increased in RA patients, although only anti-BiP antibodies were slightly increased in SLE patients. Interestingly, anti-citBiP antibody levels were higher than anti-BiP antibody levels in 72% of RA patients, whereas no significant increase in anti-citBiP antibody levels was detected in SLE patients and healthy controls. The serum levels of anti-CCP antibodies were correlated with those of anti-citBiP antibodies in RA patients (R^2 ^= 0.41). Several citrulline residues of citBiP were determined to be major epitopes of anti-citBiP antibodies, one of which showed cross-reactivity with CCP. Immunization of DBA/1J mice with citBiP induced several kinds of ACPAs, including anti-CCP and anti-citrullinated fibrinogen antibodies. Pre-immunization with citBiP exacerbated CIA, and anti-CCP antibody levels were increased in citBiP-pre-immunized CIA mice.

**Conclusions:**

CitBiP is a newly described ACPA target that may play a pro-inflammatory role in arthritis.

## Introduction

Rheumatoid arthritis (RA) is described as a chronic inflammation of multiple joints with destructive processes and is characterized by sustained synovitis, pannus proliferation, and destruction of the cartilage and bones. Many inflammatory processes participate in the pathogenesis of RA, and autoimmune responses are considered fundamental abnormalities in RA [1]. Autoantibodies such as rheumatoid factor (RF) are detected in the serum and synovial fluid of RA patients. Although the sensitivity of RF in diagnosing RA is 30%-70% in early cases and 80%-85% in progressive cases, the specificity of RF is ~40% [2]. Recently, anti-citrullinated protein/peptide antibodies (ACPAs) were reported to be highly specific in the diagnosis of RA [3,4]. Detection systems for anti-cyclic citrullinated peptide (CCP) antibodies have been improved, and the sensitivity and specificity of anti-CCP antibodies in the diagnosis of RA are 60%-80% and 95%-98%, respectively [5,6]. Importantly, anti-CCP antibodies are detected several years before joint inflammation is observed [7,8]. Due to the high specificity of ACPAs in RA, their role in the pathogenesis of RA has become the focus of active investigation.

ACPAs were first described as anti-rat esophageal antibodies, and Girbal-Neuhauser et al. discovered that citrullinated filaggrin was a target antigen of those antibodies [3]. Although citrullinated filaggrin is not present in the inflammatory synovium of RA patients, several citrullinated auto-antigens, including citrullinated fibrinogen, vimentin, type II collagen, and alpha-enolase, have been reported as target antigens of ACPAs in the synovia of RA patients [9-14]. In one hypothesis for the pathogenesis of RA, ACPAs bind to these citrullinated auto-antigens in the synovial tissues and form immune complexes that induce inflammatory processes [15]. Once synovial inflammation occurs, apoptosis and protein citrullination are induced. The continuous production of ACPAs and immune complexes results in sustained joint inflammation [16]. Indeed, serum C1q-binding immune complexes isolated from RA patients contained citrullinated fibrinogen [17], and immunization of citrullinated fibrinogen induced inflammatory arthritis in HLA-DR4 transgenic mice [18]. However, the mechanisms by which ACPAs develop and synovial proteins are citrullinated in humans remain unclear. Furthermore, the causes of RA remain unclear; it is suggested that genetic and environmental factors could cause RA, and several genetic risk factors have recently been determined. Importantly, single nucleotide polymorphisms in the peptidylarginine deaminase, type IV (PADI4) gene, which encodes a key enzyme for protein citrullination, are associated with RA susceptibility [19]. Therefore, auto-antigen citrullination and ACPA development are considered as important steps in the pathogenesis of RA.

The presence of serum anti-immunoglobulin binding protein (BiP) antibodies has been reported in RA sera, and anti-BiP antibodies showed similar sensitivity and specificity as RF [20,21]. BiP is a member of the heat shock protein 70 family and is expressed in the endoplasmic reticulum. It functions as a molecular chaperone and can bind to many proteins. BiP concentrations are elevated in the synovial fluid of RA patients [21], and BiP-responsive T cells are also detected in RA patients [22]. Here, we described the detection of anti-citrullinated BiP (citBiP) antibodies in the serum of RA patients. An epitope mapping study revealed that several citrulline residues were recognized by anti-citBiP antibodies. In a mouse study, we observed that immunization with citBiP induced ACPAs, including anti-CCP and anti-citrullinated fibrinogen antibodies. In addition, collagen-induced arthritis (CIA) was exacerbated by pre-immunization with citBiP. Therefore, we concluded that citBiP is a newly determined target of ACPAs and that it is closely related to the pathogenesis of inflammatory arthritis.

## Materials and methods

### Patients

Serum samples were obtained from 100 RA patients, 60 systemic lupus erythematosus (SLE) patients, and 30 healthy volunteers. All of the RA patients fulfilled the 1987 and 2010 American College of Rheumatology criteria for RA [23,24]. SLE diagnosis was made according to the diagnostic criteria of the American College of Rheumatology [25,26]. Written informed consent was obtained before blood samples were taken. This study was approved by the ethics committee of Tokyo University Hospital.

### Mice

DBA/1J female mice (age: 6-8 weeks) were obtained from SLC Japan (Shizuoka, Japan). All animal experiments were conducted in accordance with institutional and national guidelines.

### Antigens and peptides

A full-length fragment of mouse BiP cDNA (GenBank: AJ002387) was subcloned, and the plasmid vector, which contained a mouse BiP cDNA sequence with a His^6^-tag at its N-terminal, was prepared using a Champion pET Directional TOPO Expressing Kit (K100-01; Invitrogen, Carlsbad, CA, USA). The vector was transduced into BL21 Star One Shot Chemically Competent *Escherichia coli *(Invitrogen) by using a heat shock procedure. The transformed BL21 *E. coli *were incubated in LB medium with ampicillin (100 μg/mL). When the absorbance (O.D. 650 nm) rose to 0.5, 1 mM of isopropylthio-beta-galactoside (Invitrogen) was added to the medium. After a 6 h incubation, the pellet was dissolved using the Fast Break Cell Lysis Reagent (Promega, Madison, WI, USA) and the lysate was purified using an Ni-NTA Fast Start Kit (QIAGEN, Valencia, CA, USA) according to the manufacturer's procedure. Mouse fibrinogen was purchased from Sigma-Aldrich (St. Louis, MO, USA). For endotoxin removal, purified proteins were passed through a Detoxi-Gel AffinityPak prepared column (Pierce, Rockford, IL, USA) according to the manufacturer's protocol.

For protein citrullination, 2 U of rabbit PADI2 (Sigma-Aldrich) were added to 1 mg of the protein in buffered medium (0.1 M Tris-HCl, 10 mM CaCl_2_, 5 mM DTT) at 37°C for 2 h. EDTA (20 mM) was added to stop the reaction [15]. The samples were then passed through the Ni-NTA column to remove PADI2.

The peptides (Tables 1 and 2) were synthesized using NeoMPS (San Diego, CA, USA). Native BiP-derived peptides were also prepared that covered the entire human BiP sequence (GenBank: CAA61201) (Table 1). BiP includes 27 arginine residues that could be replaced by citrulline; therefore, we designed 27 synthesized peptides that each contained 1 citrulline residue (Table 2). In some experiments, we also prepared original peptides without citrullination. Peptide purity was >80%.

**Table 1:** Synthetic peptides derived from native human BiP protein

Number	Amino acid sequences	Position
BiP1	EDKKEDVGTVVGIDLGTTYS	21-40
BiP2	GTTYSCVGVFKNGRVEIIAN	36-55
BiP3	EIIANDQGNRITPSYVAFTP	51-70
BiP4	VAFTPEGERLIGDAAKNQLT	66-85
BiP5	KNQLTSNPENTVFDAKRLIG	81-100
BiP6	KRLIGRTWNDPSVQQDIKFL	96-115
BiP7	DIKFLPFKVVEKKTKPYIQV	111-130
BiP8	PYIQVDIGGGQTKTFAPEEI	126-145
BiP9	APEEISAMVLTKMKETAEAY	141-160
BiP10	TAEAYLGKKVTHAVVTVPAY	156-175
BiP11	TVPAYFNDAQRQATKDAGTI	171-190
BiP12	DAGTIAGLNVMRIINEPTAA	186-205
BiP13	EPTAAAIAYGLDKREGEKNI	201-220
BiP14	GEKNILVFDLGGGTFDVSLL	216-235
BiP15	DVSLLTIDNGVFEVVATNGD	231-250
BiP16	ATNGDTHLGGEDFDQRVMEH	246-265
BiP17	RVMEHFIKLYKKKTGKDVRK	261-280
BiP18	KDVRKDNRAVQKLRREVEKA	276-295
BiP19	EVEKAKRALSSQHQARIEIE	291-310
BiP20	RIEIESFYEGEDFSETLTRA	306-325
BiP21	TLTRAKFEELNMDLFRSTMK	321-340
BiP22	RSTMKPVQKVLEDSDLKKSD	336-355
BiP23	LKKSDIDEIVLVGGSTRIPK	351-370
BiP24	TRIPKIQQLVKEFFNGKEPS	366-385
BiP25	GKEPSRGINPDEAVAYGAAV	381-400
BiP26	YGAAVQAGVLSGDQDTGDLV	396-415
BiP27	TGDLVLLDVCPLTLGIETVG	411-430
BiP28	IETVGGVMTKLIPRNTVVPT	426-445
BiP29	TVVPTKKSQIFSTASDNQPT	441-460
BiP30	DNQPTVTIKVYEGERPLTKD	456-475
BiP31	PLTKDNHLLGTFDLTGIPPA	471-490
BiP32	GIPPAPRGVPQIEVTFEIDV	486-505
BiP33	FEIDVNGILRVTAEDKGTGN	501-520
BiP34	KGTGNKNKITITNDQNRLTP	516-535
BiP35	NRLTPEEIERMVNDAEKFAE	531-550
BiP36	EKFAEEDKKLKERIDTRNEL	546-565
BiP37	TRNELESYAYSLKNQIGDKE	561-580
BiP38	IGDKEKLGGKLSSEDKETME	576-595
BiP39	KETMEKAVEEKIEWLESHQD	591-610
BiP40	ESHQDADIEDFKAKKKELEE	606-625
BiP41	KELEEIVQPIISKLYGSAGP	621-640
BiP42	GSAGPPPTGEEDTAELHHHH	636-655

BiP = immunoglobulin-binding protein.

**Table 2:** Synthetic peptides

Number	Amino acid sequences	Position
1	YSCVGVFKNG(Cit)VEIIANDQG	39-58
2	VEIIANDQGN(Cit)ITPSYVAFT	50-69
3	SYVAFTPEGE(Cit)LIGDAAKNQ	64-83
4	NPENTVFDAK(Cit)LIGRTWNDP	87-106
5	TVFDAKRLIG(Cit)TWNDPSVQQ	91-110
6	TVPAYFNDAQ(Cit)QATKDAGTI	171-190
7	AGTIAGLNVM(Cit)IINEPTAAA	187-206
8	AAAIAYGLDK(Cit)EGEKNILVF	204-223
9	THLGGEDFG(Cit)VMEHFIKLY	251-270
10	LYKKKTGKDV(Cit)KDNRAVQKL	269-288
11	KTGKDVRKDN(Cit)AVQKLRREV	273-292
12	RKDNRAVQKL(Cit)REVEKAKRA	279-298
13	KDNRAVQKLR(Cit)EVEKAKRAL	280-299
14	KLRREVEKAK(Cit)ALSSQHQAR	287-306
15	AKRALSSQHQA(Cit)IEIESFFE	295-314
16	FEGEDFSETLT(Cit)AKFEELNM	313-332
17	AKFEELNMDLF(Cit)STMKPVQK	325-344
18	IDEIVLVGGST(Cit)IPKIQQLV	356-375
19	VKEFFNGKEPS(Cit)GINPDEAV	375-394
20	TVGGVMTKLIP(Cit)NTVVPTKK	428-447
21	TVTIKVYEGE(Cit)PLTKDNHLL	460-479
22	FDLTGIPPAP(Cit)GVPQIEVTF	482-501
23	TFEIDVNGIL(Cit)VTAEDKGTG	500-519
24	NKITITNDQN(Cit)LTPEEIERM	522-541
25	QNRLTPEEIE(Cit)MVNDAEKFA	530-549
26	FAEEDKKLKE(Cit)IDTRNELES	548-567
27	EDKKLKERIDT(Cit)NELESYAYS	551-570
		
11R	KTGKDVRKDNRAVQKLRREV	273-292
12R	RKDNRAVQKLRREVEKAKRA	279-298
15R	AKRALSSQHQARIEIESFFE	295-306
23R	TFEIDVNGILRVTAEDKGTG	500-519

Cit: citrulline residue.

### Immunization of mice

Murine BiP and citBiP were emulsified with an equal volume of complete Freund's adjuvant (CFA) (Chondrex, Redmond, WA, USA), and DBA/1J mice were immunized with 100 μg of murine BiP or citBiP intradermally at the tail base. Spleen samples for culture were obtained at 14 days post-immunization and serum samples for antibody titer measurements were obtained at 28 days post-immunization.

### ELISA

The serum levels of the anti-CCP antibodies were measured using MESACUP FCCP200 (Axis-Shield Diagnostics Ltd., Dundee, UK). Anti-CCP antibodies in the human serum samples were measured according to the manufacturer's protocol. Goat anti-mouse IgG-alkaline phosphate (Chemicon, Temecula, CA, USA) was used at a 3000-fold dilution as the detection antibody for measuring mouse serum anti-CCP antibodies [15]. In the competitive assay, the indicated concentrations of citrullinated peptides were added to human anti-CCP antibody-positive standard serum samples (a mixture of serum from 18 ACPA-positive RA patients), the samples were incubated for 2 h at room temperature, and the anti-CCP antibody levels were then measured.

To detect antibodies to other proteins, 5 μg/mL of each antigen was dissolved in 0.1 M NaHCO_3 _and 100 μL of each were plated in 96-well plates (Immuron4 Multiplate; Dynatech Laboratories, Chantilly, VA, USA) at 4°C for 16 h. After the plates were blocked using PBS containing 3% skimmed milk and 0.5% Tween-20, ×100 diluted samples were applied. Goat anti-human IgG-horseradish peroxidase (HRP) (Vector, Burlingame, CA, USA) or goat anti-mouse IgG-HRP antibodies (Zymed, South San Francisco, CA, USA) were used at a 5000-fold dilution as detection antibodies and TMB solution (KPL, Gaithersburg, MD, USA) was used to develop the colors. An automatic microplate reader (Bio-Rad 550; Bio-Rad, Hercules, CA, USA) was used to measure optical density.

### Immunoblotting

After 1 μg BiP or citBiP was electrophoresed using sodium dodecyl sulfate-polyacrylamide gel electrophoresis, the samples were blotted to Hybond ECL (GE Healthcare). The membranes were blocked with PBS containing 10% skimmed milk at room temperature for 1 h and ×100 diluted serum samples were then applied at 4°C for 16 h. A goat anti-human IgG-HRP antibody was used at a 5000-fold dilution as the detection antibody. The membranes were treated with ECL reagent (GE Healthcare, Buckinghamshire, UK) and the film images were developed. A polyclonal rabbit anti-BiP antibody (Thermo Fisher Scientific, Rockford, IL, USA) was used at a 100-fold dilution as a positive control. A Senshu antibody (rabbit polyclonal anti-modified citrulline antibody; Upstate Biochemicals, Lake Placid, NY, USA) was used to detect the citrullinated antigens, and blotting was performed in accordance with the manufacturer's protocol [27]. Band density was quantified using NIH ImageJ software.

### Immunofluorescence assay

Rat esophageal sections (SCIMEDX Corporation, Denville, NJ, USA) were used, and citrullinated filaggrin was detected according to the manufacturer's protocol. In short, mouse serum samples were treated at room temperature for 1 h and Alexa Fluor 488-conjugated goat anti-mouse IgG antibody (Invitrogen) was used as the detection antibody. The samples were observed under a fluorescent microscope [15].

### Collagen-induced arthritis

CIA was induced as described previously [28]. In short, bovine type II collagen (Chondrex) was emulsified with an equal volume of CFA or incomplete Freund's adjuvant (IFA) (Chondrex). Fourteen days after immunization with BiP or citBiP, DBA/1J mice were immunized with 50 μg bovine type II collagen intradermally at the tail base on day 14 with CFA and on day 35 with IFA. The arthritis score was determined by the degree of erythema, swelling, or ankylosis observed on each paw, as described elsewhere [29]. The mice were sacrificed at 50 days after the first CIA immunization. Tissue samples were embedded in paraffin wax after 10% formaldehyde fixation and decalcification. The sections were stained with hematoxylin and eosin. Synovial tissues were graded by mononuclear cell infiltration, pannus formation, and cartilage erosion, as described previously [30].

### Statistical analysis

All values are expressed as the median. Differences were compared using the Mann-Whitney U test. Correlations between the levels of two antibodies were analyzed by the Spearman correlation coefficient. Graphpad Prism 5 was used for statistical analysis. *P*-values < 0.05 were considered significant.

## Results

### Anti-citBiP antibodies in the serum of RA patients

**Figure 1:**
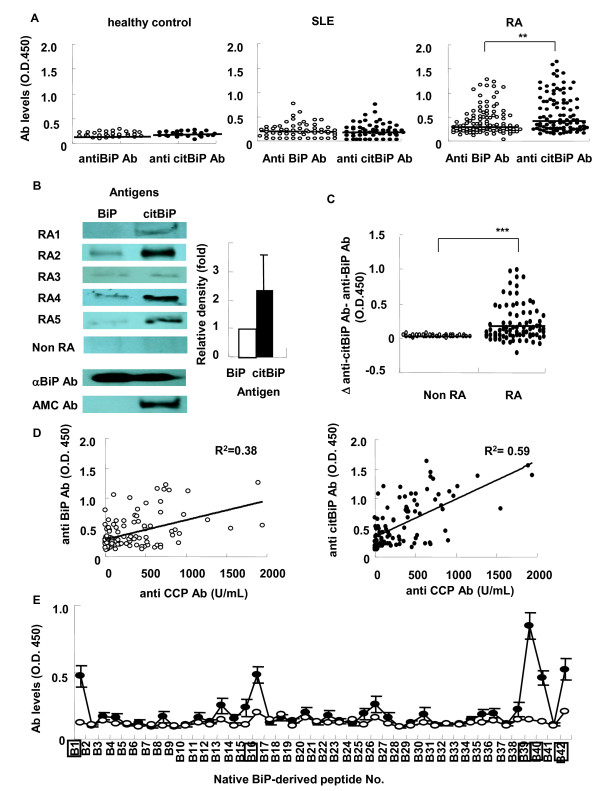
**Serum anti-citrullinated BiP (citBiP) antibodies were detected in rheumatoid arthritis (RA) patients at a high frequency, but not in systemic lupus erythematosus (SLE) patients or healthy controls**. **A**. Comparison of the levels of anti-BiP and citBiP antibodies in healthy controls (n = 30), SLE patients (n = 60), and RA patients (n = 100). The serum levels of anti-BiP and citBiP antibodies were measured by ELISA. **: *P *< 0.01. **B**. Immunoblotting of the representative serum samples from anti-citBiP antibody-positive RA patients. AMC stands for anti-modified citrulline antibody (Senshu antibody). Band densities were quantified by ImageJ, and the fold increase is indicated. **C**. The difference between the serum levels of anti-citBiP and anti-BiP antibodies in the serum of RA patients and non-RA controls. ***: *P *< 0.001. **D**. Correlation between anti-CCP and anti-BiP/citBiP antibodies. **E**. Epitope mapping of anti-BiP antibodies. The serum levels of antibodies to native BiP-derived peptides. The sequences of the native BiP-derived peptides are shown in Table 2. The serum samples were taken from anti-BiP antibody-positive RA patients (n = 15, closed circles) and healthy controls (n = 5, open circles). The horizontal lines indicate medians. P-values were calculated by Mann-Whitney U test.

We first measured the serum levels of anti-BiP and anti-citBiP antibodies in RA patients, SLE patients, and healthy controls (Figure 1A). Among these patients, 95 RA patients and no SLE patients were positive for anti-CCP antibodies. We chose SLE patients as disease controls because ~30% of SLE patients are positive for anti-BiP antibodies, while serum anti-CCP antibodies are detected in <5% of SLE patients [31]. In RA patients, the levels of anti-BiP and anti-citBiP antibodies were significantly higher than those observed in SLE patients and healthy controls (*p *< 0.001). Immunoblotting analysis demonstrated higher serum concentrations of anti-citBiP antibodies than anti-BiP antibodies in the serum of each anti-CCP antibody-positive RA patient (Figure 1B). Seventy-two percent of RA patients showed higher anti-citBiP antibody levels than anti-BiP antibody levels, whereas there was no significant difference in the serum titers of anti-BiP and anti-citBiP antibodies in the non-RA controls (Figure 1C). A previous report suggested that serum anti-BiP antibodies were detected in SLE patients [31]; in our study, the serum levels of anti-BiP and anti-citBiP antibodies in SLE patients were mildly increased in comparison with those in healthy controls (*p *< 0.05) (Figure 1A). However, in contrast to RA, there was no significant difference in the serum levels of anti-BiP and anti-citBiP antibodies in SLE patients (Figure 1C). In RA patients, the serum levels of anti-citBiP antibodies were strongly correlated with those of anti-CCP antibodies (R^2 ^= 0.59), whereas the levels of anti-BiP antibodies were less correlated with those of anti-CCP antibodies (R^2 ^= 0.38) (Figure 1D). Therefore, the serum levels of anti-citBiP antibodies are higher than those of anti-BiP antibodies and have a clear association with anti-CCP antibodies in RA patients.

Because BiP is an immunoglobulin chaperon protein, there is the possibility that immunoglobulins bind to BiP, independent of epitope recognition by the antigen-binding site. To confirm the existence of specific epitope recognition, epitope mapping was performed by using native human BiP-derived peptides. The mapping revealed that BiP1 (BiP_21-40_), BiP16 (BiP_246-265_), BiP39 (BiP_591-610_), BiP40 (BiP_606-625_), and BiP42 (BiP_636-655_) were the epitopes recognized by the anti-BiP antibodies (Figure 1E). Most of these major epitopes were located in the C and N terminals. Therefore, anti-human BiP antibodies truly exist in the serum of RA patients.

### Determination of the citrullinated epitopes of anti-citBiP antibodies

**Figure 2:**
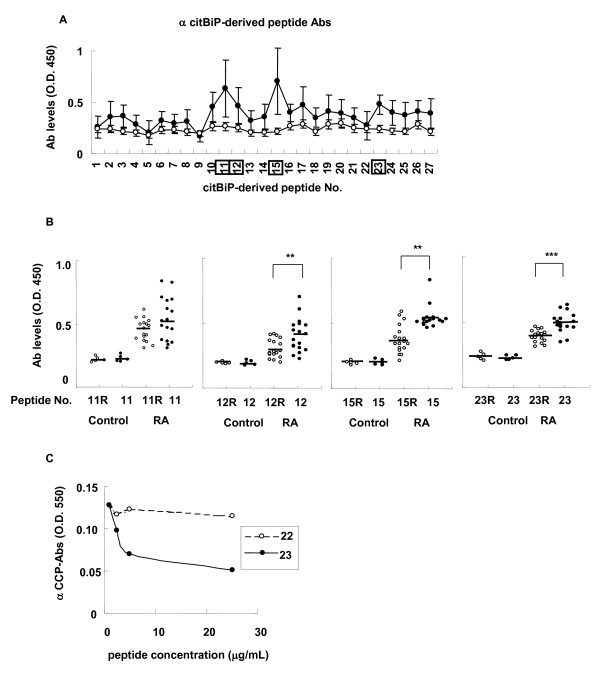
**CitBiP-derived epitopes were identified by epitope mapping and one of them exhibited cross-reactivity to cyclic citrullinated peptide (CCP)**. **A**. The serum levels of antibodies to cit-BiP-derived citrullinated peptides. The serum samples were taken from anti-citBiP antibody-positive rheumatoid arthritis (RA) patients (n = 18, closed circle) and healthy controls (n = 5, open circle). **B**. Comparison between the serum levels of anti-native peptide antibodies (open circles) and citrullinated peptide antibodies (closed circles). (RA, n = 18; healthy controls, n = 5) **: *P *< 0.01, ***: *P *< 0.001. **C**. Competitive assay of anti-CCP antibodies from RA patients with a BiP-derived citrullinated peptide. The horizontal lines indicate medians. P-values were calculated by Mann-Whitney U test.

To identify the citrullinated epitopes of the anti-citBiP antibodies in RA, we prepared citrullinated peptides in which the arginine residues of the native BiP sequences were replaced with citrulline residues. Serum antibody levels to each of the citBiP-derived peptides were measured in RA patients with high anti-citBiP antibody levels (n = 18) and healthy controls (n = 5). The serum antibody levels were increased against several citrullinated peptides (Figure 2A). Four of the major citBiP-derived epitopes were selected for further investigation, and the serum levels of the anti-native peptide antibodies and the anti-citrullinated peptide antibodies were compared (Figure 2B). The serum antibody levels to No. 12 (BiP_279-298 _R289citrulline), 15 (BiP_295-314 _R305citrulline), and 23 (BiP_500-519 _R510citrulline) citrullinated peptides were significantly increased compared with those of the native peptides in RA patients. Thus, these citrullinated residues were thought to be important residues for autoantibody recognition in RA. Thus, the positions of the major citrullinated epitopes (BiP_279-298_, BiP_295-314_, and BiP_500-519_) were different from the major epitopes of the native BiP protein (BiP_21-40_, BiP_246-265_, BiP_591-610_, BiP_604-625_, and BiP_636-655_). In particular, the No. 23 citrullinated peptide inhibited serum antibody binding to CCP, whereas the No. 22 peptide did not (Figure 2C). No. 12 and 15 citrullinated peptides did not bind to CCP either (data not shown). Therefore, we identified several citrullinated anti-citBiP antibody epitopes, one of which showed a clear cross-reaction with CCP.

### Induction of ACPAs in citBiP-immunized mice

**Figure 3:**
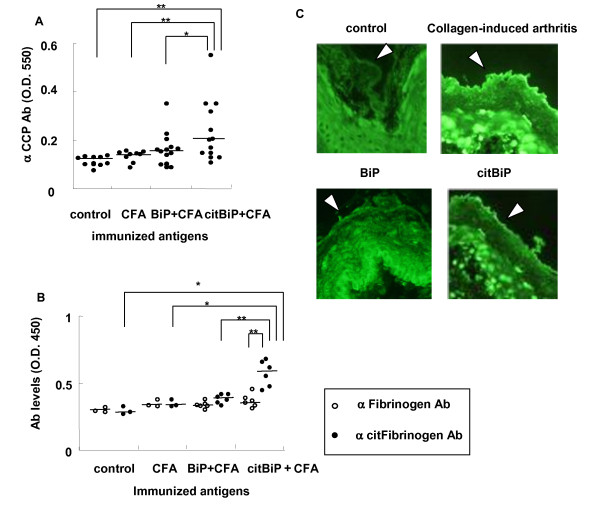
**Immunization of citBiP induced anti-citrullinated protein/peptide antibodies in mice**. Immunized citBiP was prepared in the same way as the one shown in Figure 1B. **A**. Serum levels of anti-CCP antibodies in control, complete Freund's adjuvant (CFA)-immunized, BiP+CFA-immunized, and citBiP+CFA-immunized mice. **B**. Serum levels of anti-fibrinogen and anti-citrullinated fibrinogen antibodies in control and immunized mice. **: *P *< 0.01. **C**. Immunofluorescence assay of mouse serum to the rat esophageal horny layer (arrows). The horizontal lines indicate medians. P-values were calculated by Mann-Whitney U test.

We then examined whether active immunization with citBiP could induce ACPAs in a mouse model. DBA/1J mice were immunized with BiP or citBiP and serum samples were obtained after 28 days. Anti-CCP antibodies were detected in the serum samples of citBiP-immunized mice (Figure 3A). Although BiP immunization also induced the development of anti-CCP antibodies, the anti-CCP antibody levels were relatively low. Because Vossenaar et al. pointed out that some mouse models of inflammatory arthritis developed false-positive anti-CCP antibodies [32], we verified the presence of ACPAs using different methods. The serum levels of anti-citrullinated fibrinogen antibodies were only detected in the sera of citBiP-immunized mice (Figure 3B). We stained rat esophageal horny layer containing citrullinated filaggrin with sera from citBiP-immunized mice (Figure 3C). Therefore, we confirmed that citBiP immunization not only induced anti-citBiP antibodies but also induced other ACPAs.

### Exacerbation of CIA by pre-immunization with citBiP

**Figure 4:**
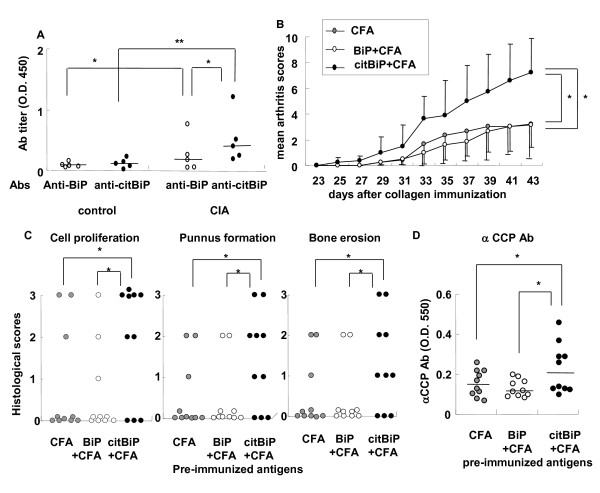
**Pre-immunization of citBiP, and not native BiP, exacerbated collagen-induced arthritis (CIA) in mice**. **A**. Serum levels of anti-BiP and anti-citBiP antibodies in control and CIA mice. Serum was obtained at 50 days after the first immunization. *: *P *< 0.05, ***: *P *< 0.001. **B**. Arthritis scores of CIA in complete Freund's adjuvant (CFA), BiP+CFA, and citBiP+CFA-pre-immunized mice (n = 10 each). *: *P *< 0.05. **C**. Histological CIA scores in CFA, BiP+CFA, and citBiP+CFA-pre-immunized mice (n = 10 each). *: *P *< 0.05. **D**. Serum levels of anti-CCP antibodies in CFA, BiP+CFA, and citBiP+CFA-pre-immunized mice (n = 10 each) after CIA development. *: *P *< 0.05, ***: *P *< 0.001. Data are representative of 3 trials and the arthritis scores for the other 2 sets of experiments are shown in Additional file 1. The horizontal lines indicate medians. P-values were calculated by Mann-Whitney U test.

Finally, we tested the pro-inflammatory effects of citBiP in the CIA mouse model. Similar to the findings in RA, CIA mouse sera contained anti-BiP and anti-citBiP antibodies (Figure 4A). The serum levels of anti-citBiP antibodies were higher than those of anti-BiP antibodies. Since the development of ACPAs precedes the onset of RA, some immune responses to citrullinated antigens are hypothesized to occur before RA becomes clinically obvious. We assessed this hypothesis by pre-immunizing mice with citBiP before inducing CIA, and determined whether the immune responses to citBiP were associated with the enhancement of inflammatory arthritis. Fourteen days after BiP or citBiP immunization, the mice were immunized with bovine type II collagen to induce arthritis. Joint inflammation developed sooner and was significantly exacerbated in the citBiP-pre-immunized mouse group as compared to the control group (Figure 4B and Additional file 1). BiP-pre-immunized mice did not develop severe arthritis. There was a significant increase in the histological scores of the citBiP-pre-immunized mouse group (Figure 4C). The serum levels of anti-CCP antibodies were also significantly elevated in the citBiP-pre-immunized mice (Figure 4D). Therefore, citBiP immunization exacerbated inflammatory arthritis in mice, whereas BiP immunization had no significant effect on CIA.

## Discussion

BiP is a molecular chaperone that is expressed in the endoplasmic reticulum and a known RA auto-antigen. BiP is overexpressed in the synovial fluids of RA patients, and anti-BiP antibodies develop in 60% of RA patients [20,21]. CD8^+ ^T cells from the peripheral blood of RA patients respond to BiP stimulation and release cytokines such as interleukin (IL)-10 [22]. In inflammatory arthritis mouse models, the intravenous administration of BiP ameliorated joint inflammation, and the Th2 cytokine IL-4 was associated with these anti-inflammatory effects [33]. Although some reports showed that BiP induces anti-inflammatory T cells that produce IL-4 and IL-10, pro-inflammatory responses to BiP were also observed. BiP-stimulated human peripheral blood mononuclear cells secreted tumor necrosis factor (TNF)-ε in the early phase [34], and mouse BiP-primed T cells simultaneously produced interferon-γ [33]. In fact, anti-BiP antibodies develop in RA patients, form immune complexes, and induce inflammatory processes in the synovium. Thus, the role of BiP in the pathogenesis of RA is controversial.

In the present study, we demonstrated that citBiP is a new target of RA autoantibodies and that the serum levels of anti-citBiP antibodies are higher in RA patients than those of anti-BiP antibodies. We performed B cell epitope mapping of BiP and citBiP. B cell epitope mapping using linear peptides sometimes misses discontinuous epitopes, which are recognized by antibodies only in the native conformation. Nevertheless, this protocol is good at identifying continuous epitopes, which are formed from single short peptide strands constructed on a generally linear backbone structure. In our study, the locations of the major epitopes of the anti-citBiP antibodies were clearly different from those of the anti-native BiP antibodies. Despite the limitation of using linear peptides for epitope mapping, these data suggested that the anti-citBiP and anti-BiP antibodies are produced via different processes in RA patients. In addition, there was no significant difference between the serum levels of anti-BiP and anti-citBiP antibodies in SLE patients, suggesting that the development of anti-citBiP antibodies was a distinct process in RA.

In the mouse model, citBiP immunization induced higher anti-CCP antibody levels than BiP immunization. Furthermore, citBiP pre-immunization exacerbated inflammatory arthritis, whereas BiP pre-immunization had no significant effects on CIA. Therefore, we believe that citBiP is more arthrogenic than BiP and could play an important role in the pathogenesis of inflammatory arthritis. In this context, the existence and localization of citBiP are important questions. Some reports have shown that BiP was overexpressed in the inflamed synovial tissues of RA patients [20,21]. Although citrullinated BiP was not detected in the synovial exosomes of RA patients [35], Lu et al. recently reported that citBiP was expressed on macrophage membranes, a target of ACPAs [36]. They also demonstrated that ACPAs could bind to citBiP on macrophage surfaces to induce the secretion of TNF-ε. Therefore, it is possible that macrophage membrane-bound citBiP is a source of the citrullinated protein in some situations.

The role of protein citrullination in the pathogenesis of RA has been widely investigated, and some reports explained the role of protein citrullination in the immune system. First, protein citrullination modifies the electric charge, i.e., arginine is positively charged, while citrulline is neutral. This electric change may result in a structural change of the protein and reveal hidden epitopes [37]. Other groups have reported that protein citrullination itself reinforced antigenicity and induced more intense immune responses. The citrullinated auto-antigens possessed enough antigenicity to break immune tolerance to the auto-antigens [18,38]. In our data, citBiP immunization induced several kinds of ACPAs and exacerbated arthritis. We suppose that the pre-induction of anti-CCP antibodies could explain this pro-arthritis effect of citBiP immunization. However, the precise role of anti-CCP antibodies in mice remains unclear. Although more observations are necessary to demonstrate how the preceding immune responses to citBiP exacerbate CIA, these studies suggested the reason why the immune responses to citBiP were different from those to BiP in RA patients and our mouse model.

In the mouse study, we demonstrated that citBiP-immunization could induce several ACPAs. One possible explanation is epitope spreading. Epitope spreading has been demonstrated in a variety of spontaneous and induced autoimmune diseases, such as experimental autoimmune encephalitis and diabetes in NOD mice [38,39]. A recent study showed that epitope spreading of ACPAs was observed in RA patients [40]. In our mouse study, immunization with citBiP could induce antibody responses to other citrullinated proteins, such as citrullinated fibrinogen and filaggrin. The pivotal role of B cells in epitope spreading has been proposed because B cells are very efficient antigen-presenting cells or antigens that are taken up specifically through the B-cell receptor [41]. On the basis of the fact that different ACPA responses are cross-reactive [42], one hypothesis suggests that B cells expressing the B-cell receptor specific to citBiP could uptake and present several kinds of citrullinated proteins other than citBiP. The immune response to citBiP immunization could be a good model to study how the development of several ACPAs occurs, although more examinations are required.

With regard to the majority of target proteins for ACPA, including fibrinogen, vimentin, and alpha-enolase, antibodies react only with the citrullinated forms of these molecules and not with their native forms. In sharp contrast, antibodies to native BiP are readily detected in RA patients [20,21]. In this study, immunization with BiP also induced low anti-CCP antibody titers. Ireland et al. suggested that APCs could present citrullinated peptides even when given an unmodified protein [43], which could be an explanation for this phenomenon. Nonetheless, subcutaneous pre-immunization with BiP coupled with adjuvant induced no significant effects on CIA development in our experiments. These results suggest that an immune response to the native form of BiP is not sufficient for the progression of arthritis and that a significant level of immunity against citBiP is required. However, immunity to BiP and citBiP may augment the production of other ACPAs in RA.

## Conclusions

In conclusion, we demonstrated that anti-citBiP antibodies appeared in the sera of RA patients. Because the serum levels of anti-citBiP antibodies were higher than those of anti-BiP antibodies in 72% of RA patients, and the location of the major epitopes of anti-citBiP and anti-BiP antibodies were different in RA patients, the development of anti-citBiP antibodies can be a distinct process in the pathogenesis of RA. In our mouse study, immunization with citBiP induced several kinds of ACPAs, including anti-CCP and anti-citrullinated fibrinogen antibodies. Furthermore, pre-immunization with citBiP exacerbated CIA. Therefore, we concluded that citBiP could play an important role in the pathogenesis of inflammatory arthritis and could be a new diagnostic and therapeutic target for RA.

## Abbreviations

ACPAs: anti-citrullinated protein/peptide antibodies; BiP: immunoglobulin binding protein; CCP: cyclic citrullinated peptide; CFA: complete Freund's adjuvant; CIA: collagen-induced arthritis; citBiP: citrullinated immunoglobulin binding protein; HRP: horseradish peroxidase; IFA: incomplete Freund's adjuvant; IL: interleukin; PADI: peptidyl arginine deiminase; RA: rheumatoid arthritis; RF: rheumatoid factor; SLE: systemic lupus erythematosus; TNF: tumor necrosis factor.

## Competing interests

The authors declare that they have no competing interests.

## Authors' contributions

HS performed the majority of the experimental study and the statistical analysis, and prepared the manuscript. KF was involved in the design of the study, collecting the patients, and helped with drafting the manuscript. MS, TO, SS, AO, TS assisted in conducting the experiments and collecting the patients. KY was involved in the design and conception of the study, and helped with drafting the manuscript. All authors read and approved the final manuscript.

## Supplementary Material

Additional file 1 **The arthritis scores for the other 2 sets of trials in collagen-induced arthritis (CIA) mice that were pre-immunized with complete Freund's adjuvant (CFA), BiP+CFA, or citBiP+CFA (n = 10 each)**. *: *P *< 0.05. Click here for file
