# Supplementary material for: Detection of autoantibodies to citrullinated BiP in rheumatoid arthritis patients and pro-inflammatory role of citrullinated BiP in collagen-induced arthritis
Source: Arthritis Res Ther. 2011 Nov 22;13(6):R191. doi: 10.1186/ar3520 (PMC3334641; doi:10.1186/ar3520)
Supplement: Additional file 1 — The arthritis scores for the other 2 sets of trials in collagen-induced arthritis (CIA) mice that were pre-immunized with complete Freund's adjuvant (CFA), BiP+CFA, or citBiP+CFA (n = 10 each). *: P < 0.05. [file ar3520-S1.PPT]

## Slide 1
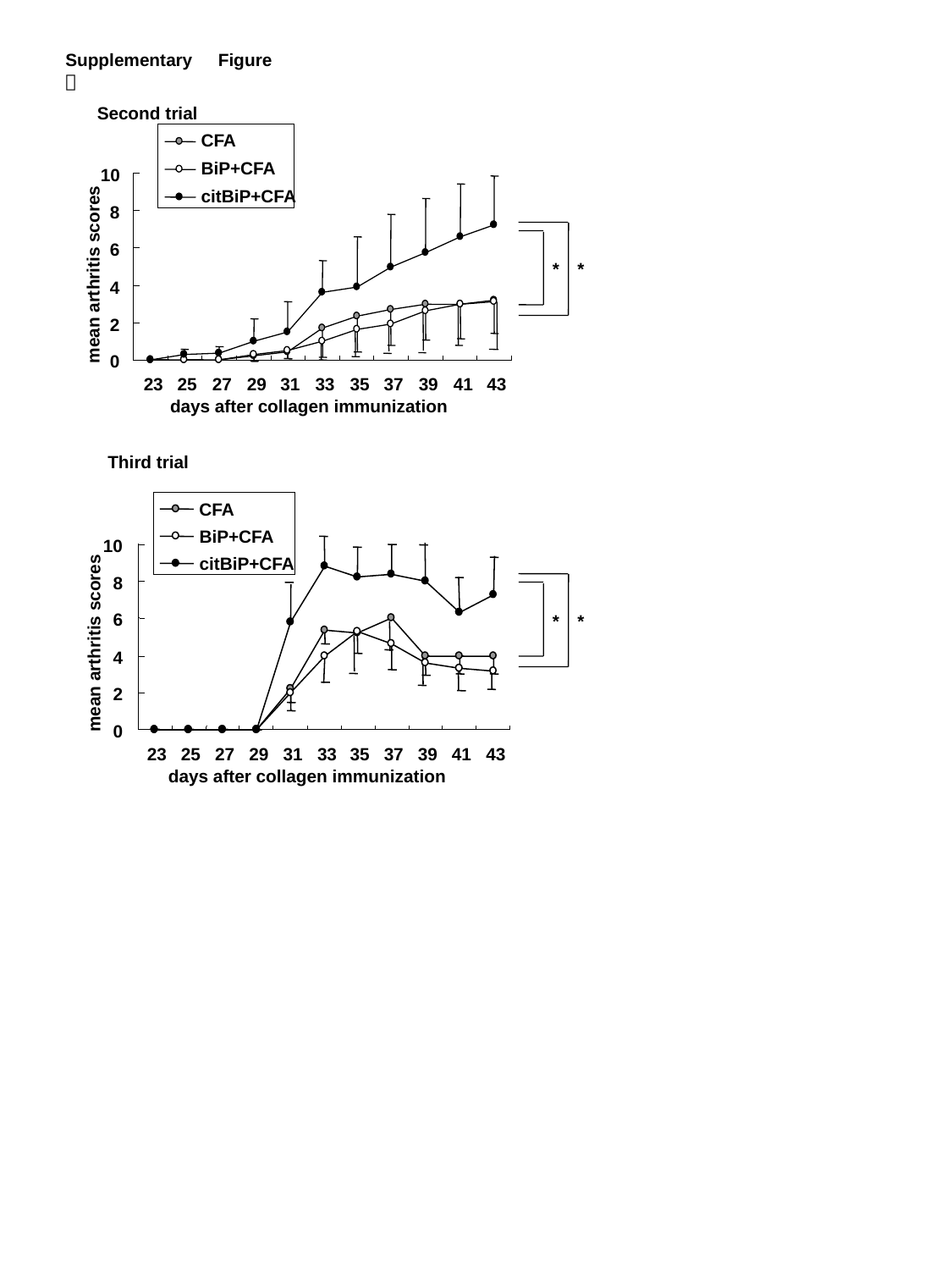

Supplementary　Figure１
Second trial
CFA
BiP+CFA
10
citBiP+CFA
8
*
*
6
mean arthritis scores
4
2
0
23
25
27
29
31
33
35
37
39
41
43
days after collagen immunization
Third trial
CFA
BiP+CFA
10
citBiP+CFA
8
*
*
6
mean arthritis scores
4
2
0
23
25
27
29
31
33
35
37
39
41
43
days after collagen immunization
